# Sickness Presenteeism in the Aftermath of COVID-19: Is Presenteeism Remote-Work Behavior the New (Ab)normal?

**DOI:** 10.3389/fpsyg.2021.748053

**Published:** 2022-01-27

**Authors:** Aristides I. Ferreira, Merce Mach, Luis F. Martinez, Mariella Miraglia

**Affiliations:** ^1^Business Research Unit, Iscte – Instituto Universitário de Lisboa, Lisbon, Portugal; ^2^Faculty of Economics and Business, University of Barcelona, Barcelona, Spain; ^3^NOVA School of Business and Economics, Universidade NOVA de Lisboa, Lisbon, Portugal; ^4^Management School, Faculty of Humanities & Social Sciences, University of Liverpool, Liverpool, United Kingdom

**Keywords:** sickness presenteeism, remote work, societal context, cross-cultural issues, COVID-19, changes in work practices

## Abstract

Due to the confinement imposed by the COVID-19 pandemic situation, companies adopted remote work more than ever. The rapid rise of remote work also affected local life and many employers introduced or extended their telework activities because of the associated advantages. However, despite the evident positive benefits, some employees were pressured to work remotely while ill. This evidence brought new challenges to the presenteeism literature. This article investigates how individual, economic/societal, and organizational/sectorial/supervisory-related variables can moderate the role of a contagious disease, such as the COVID-19, in explaining presenteeism behavior. Moreover, the current research presents a multi-level conceptual model (i.e., organizational, individual, supervisory factors) to describe how a new construct of remote-work presenteeism behavior mediates the relationship between different post pandemic health conditions (e.g., allergies, back pain, depression, anxiety) and future cumulative negative consequences. The authors suggested that the widespread pervasive adoption of remote work because of COVID-19 has important implications for the presenteeism literature and opens avenues for further research.

## Introduction

COVID-19 has dramatically affected workers and organizations around the globe (c.f., Salem et al., 2021). Individuals have faced great challenges in terms of their health and wellbeing, changes in work practices arising with local lockdowns primarily related to the imposition of remote working, the need to strike a balance between the work and family/life domains, and the rise of unemployment, furlough schemes, and job insecurity, among others. At the same time, organizations have had to rapidly re-organize their workflows and processes, alter their human resource practices, modify operations profoundly, and find new ways to lead and motivate remote workers and teams. Moreover, businesses have been struggling to maintain productivity and profits in the face of the economic crisis associated with the pandemic. Similarly, the call to reduce costs associated with sickness absenteeism (Kinman and Grant, 2020) along with the related costs of increased presenteeism, which is to say, working when sick (Aronsson et al., 2000) has influenced this new working environment. This might seem paradoxical in the context of a pandemic, in which the concept of health moves to the fore and the risks associated with attending work while sick are evident, considering the threat of contagion (Pichler and Ziebarth, 2017) and the spread of the virus in the workplace. Thus, presenteeism is no longer an individual behavior – a personal choice between going to work or not in the face of illness. Instead, it now configures as a behavior that is potentially dangerous for workplace, making it a shared issue, a public health matter.

To reflect this renewed collective meaning associated with the construct, the present investigation moves away from a focus on the individual determinants of presenteeism and seeks to draw attention to some of the most important organizational, occupational, and societal factors that can impact working while sick during the COVID-19 pandemic. This shift is also in line with calls in the literature to fully consider the social determinants of attendance at work (e.g., Ruhle et al., 2020; Miraglia and Johns, 2021).

Our conceptual study first examines the organizational factors that may lead to presenteeism in the context of COVID-19 by illustrating how changes in working practices triggered by the pandemic – specifically, remote working, remote teamwork, and remote leadership – affect working while sick, as well as the role that presenteeism climate (Ferreira et al., 2015) plays in encouraging or discouraging the behavior. The study will then examine variation in presenteeism across occupational sectors during COVID-19. Finally, the societal context will be considered, investigating how a country’s legislative context (e.g., welfare and social security systems, and work regulations), its labor market, economic conditions, and its cultural values may prompt working while sick during COVID-19, both directly and through shaping people’s notions of health. Also, differences in presenteeism behaviors during COVID-19 will be explained via looking at poverty, precarious work, and inequality, which are highlighted by the pandemic.

Moreover, the study attempts to guide future research and practice on presenteeism by proposing two models. First, by drawing on event system theory (Morgeson et al., 2015) and on the pivotal model by Johns (2010) regarding the factors intervening in the relationship between health conditions (i.e., acute, episodic, or chronic) and the individual choice between absenteeism and presenteeism, we build a model that accounts for acute and contagious health conditions (i.e., contracting the virus) as well as individual, economic/societal, and occupational/sectorial factors to explain presenteeism behaviors. Second, we propose the concept of remote-work presenteeism behavior, whereby continuing to work when sick while at home may become normalized, pressuring individuals into it. We discuss the abnormality of this in the light of the COVID-19 pandemic context and the negative consequences that presenteeism bring for individuals and organizations (Evans-Lacko and Knapp, 2016; Skagen and Collins, 2016). We conclude with a second model identifying the societal, organizational, supervisory, and individual dimensions that can foster such behaviors.

## The Organizational and Occupational Context

### Remote Working

The COVID-19 pandemic has thrust upon us new ways of working, first by causing a rapid shift to “forced” remote work, especially for knowledge-intensive and office-based workers. The so-called “work-from-home experiment” (Harford, 2020) was enabled by the advancement in digital technologies, which allow employees to communicate, share data, and collaborate on projects and documents in real-time via audio, video, and/or text means. The shift to remote work has clear repercussions on the behavior of presenteeism and can also accelerate the adoption of digital practices, such as electronic monitoring and appraisal, which have important implications for presenteeism research.

The use of digital technologies associated with remote work creates an increased amount of employees’ work inputs and outputs that are recorded and stored by the organization (Leonardi, 2021). When combined, these meta-data, also known as digital exhaust, can create “digital footprints” that reveal workers’ patterns and can became a powerful instrument to monitor behavior, inputs, and outcomes at work (Leonardi, 2021). In the context of remote working, managers can use time tracking systems, which record the time spent on specific job activities (e.g., time spent on applications, keystrokes, emails read), or pervasive tracking, which keeps an open, continuous communication channel (e.g., having cameras on during the working day, being constantly connected to a chat app) with the employee, to monitor the productivity of remote workers (Nguyen, 2020).

Through the use of such control technologies, the digitalization of work enabled by remote working may make it more feasible for organizations to implement electronic monitoring and intrusive surveillance (George et al., 2020). This has implications for attendance behavior, including presenteeism. First, when employees recognize that their online activities generate digital footprints that can be followed by their employers, they may perceive their inputs and activities as extremely visible and under scrutiny, and therefore put in greater effort, which has been found to result in greater burnout (Cristea and Leonardi, 2019). By intensifying the employees’ efforts at work (Delbridge et al., 1992), monitoring can promote a culture of being constantly available (Parker et al., 2020) and generate feelings of attendance pressure, likely leading to working when sick (Aronsson and Gustafsson, 2005; Baker-McClearn et al., 2010).

Second, as electronic monitoring of employee performance is perceived as intense and controlling (Miller, 2003; Levy et al., 2017), it can directly diminish employee wellbeing (Holman et al., 2002). In fact, the perceived intensity of monitoring has been associated to greater anxiety, depression, exhaustion, and job dissatisfaction (Holman et al., 2002). Similarly, preliminary evidence from a study conducted among employees working from home during COVID-19 showed that high levels of strict monitoring caused greater anxiety at work (Parker et al., 2020). Thus, monitoring and controlling technologies can trigger presenteeism by affecting one of its deepest roots, that is, health (McGregor et al., 2018). Indeed, meta-analyses have reported negative effect of working while ill on mental health (McGregor et al., 2018), including emotional exhaustion and depression (Miraglia and Johns, 2016).

### Remote Teamwork

Although remote teamwork was an emergent phenomenon and already underway in the pre-pandemic world, COVID-19 has accelerated the shift from in-person to virtual teams (Kniffin et al., 2021). Despite offering new opportunities (e.g., better brain storming; DeRosa et al., 2007), team virtuality poses some challenges for employees, teams, and organizations (for reviews, see Kirkman et al., 2012; Mak and Kozlowski, 2019).

Relevant to presenteeism research is the effect of team virtuality on identification in organizations, which may be threatened by the increasing virtualization of work (Ashforth, 2020). For example, individual perceptions of virtuality have been reported to influence organizational identification negatively (Sohrabi et al., 2011), and unevenly geographically dispersed teams seem to experience lower team identification (O’Leary and Mortensen, 2010). In the workplace, identification can foster the introjection of norms, and the consequent adoption of shared behaviors – and this is equally true for attendance behaviors. Drawing on social influence theories, individuals from the same social unit can adhere to the predominant norm and behave in line with the expected standards to seek social approval, a sense of identity, enhanced self-esteem (in line with social identity theory; Tajfel and Turner, 1986), or to obtain information to reduce ambiguity and facilitate judgment (consistent with social information theory; Salancik and Pfeffer, 1978).

The operating of such a normative mechanism has been well documented in the absenteeism literature, whereby employees model their absentee behaviors on those of their work group or colleagues (for a review, see Miraglia and Johns, 2021). Although virtually no empirical literature exists on presenteeism norms, some evidence of their influence on individual behaviors comes from research showing that the shared team perceptions of concern about health issues reduce working when sick (Schulz et al., 2017). Other studies focusing on presenteeism climate – which can encourage presenteeism behavior – showed that the social context of presenteeism climates where key variables (such as co-workers competitiveness, extra-time valuation, and difficulty of replacement) influence the relationship between job resources (e.g., degree of autonomy at work) and the occurrence of presenteeism behavior (Mach et al., 2018).

The challenges raised by remote working and COVID-19 are to understand how identification with the norms of their context develops in virtual working environments, whether new *foci* of identification emerged, and what the impact is on attendance norms and subsequent absenteeism and presenteeism behaviors. According to event system theory (Morgeson et al., 2015), “major crises are moral inflection points because they implicitly call upon organizations to rise to the occasion by doing the right things for the greater good” (Ashforth, 2020, p. 1764). For instance, if due to the pandemic crisis, the occupational/role identity becomes more prominent (for the sake of the “greater good”) than the organizational or team one (Ashforth, 2020), individuals may adhere to the occupational norms regulating attendance (Miraglia and Johns, 2021), which in some professions (e.g., in human service organizations) can provoke working when ill (Gosselin, 2018). If the personal or “me” identity (Ashforth, 2020) becomes more salient, we could expect individual factors to have a stronger impact on presenteeism. Among others (for a review of presenteeism correlates see Lohaus and Habermann, 2019), an individual’s physical or mental health conditions (e.g., allergies, back pain, depression, anxiety), financial situation, lifestyle, and positive attitudes toward the job and the organization could have a major role in determining the individual decision of working while ill, regardless of organizational or team norms.

Working in virtual teams, coupled with the associated loss of face-to-face, daily interactions among coworkers, may make it more difficult to ask for help (Kniffin et al., 2021), having consequences on the level of social support remote employees experience. In the remote working environment imposed by the pandemic, social support has been identified as a key factor in reducing loneliness, work-family conflict (WFC), and procrastination among physically distanced employees (Wang et al., 2021). In relation to presenteeism, social support is a deterrent to working while sick (Miraglia and Johns, 2016; McGregor et al., 2018), as supportive colleagues can ease disclosure of illness in the workplace (Munir et al., 2005), legitimizing absence and decreasing attendance pressure. Moreover, collegial support figures as a job resource (Bakker et al., 2014), which reduces work-related stress and physical health symptoms (Väänänen et al., 2003). This, in turn, can be expected to diminish the incidence of presenteeism. Therefore, unless organizations set best practices to help individuals to seek and offer help and support remotely, we could predict an increase in continuing to work while sick in the remote working context.

### Remote Leadership

COVID-19 has greatly transformed leadership, forcing many leaders to rapidly transition to remote management, which can have consequences for individuals’ presenteeism behaviors. As already documented in the absenteeism (Løkke Nielsen, 2008; Duff et al., 2014) and wellbeing (Inceoglu et al., 2018) literatures, initial empirical evidence shows that employees’ presenteeism behaviors are modeled against those of the leader (Dietz et al., 2020). Supervisors have a crucial role in establishing presenteeism levels, especially during a pandemic, when health is a significant and delicate concern. In virtual teams, where communication richness is limited (Martins et al., 2004), it is essential for leaders to model healthy behaviors along with clarifying expectations and policies around sickness and attendance and promoting boundary-setting and mechanisms for switching off from work (including online communication) when sick (Kinman and Grant, 2020). Obviously, this should be accompanied by structural changes in the welfare system (e.g., offering paid sick leave) to ensure that constraints on absenteeism are lifted and presenteeism is not encouraged (Miraglia and Johns, 2016).

We could assume that the limited communication cues in a remote environment may also hinder supervisors from noticing presenteeism episodes among employees. However, a recent survey conducted by the Chartered Institute for Personnel Development (Chartered Institute for Personnel Development [CIPD], 2021) among 668 HR professionals in November/December 2020 reported that employers signaled that working while sick remained common during the pandemic, with 77% of employees working from home showing some signs of the behavior. Unanswered questions to address include how supervisors can realize when employees continue to work from home despite sickness and, more generally, how can they check employees’ health and wellbeing without encroaching on their privacy rights (Kniffin et al., 2021). Another issue to address has to do with the measures and interventions that can be put in place to tackle presenteeism in remote working. This last issue is also important in light of the above-mentioned Chartered Institute for Personnel Development [CIPD] (2021) report, which reveals that two-fifths of employers experiencing presenteeism issues among their workforce are not taking any action to address or prevent it.

### Presenteeism Climate

Presenteeism climate is another important variable that can affect individual attendance behavior. This concept is often mentioned in the literature. It results from beliefs and values about the sector, department, organization, and society that compel employees to attend work despite being ill. However, it has not been systematically measured until recently. Ferreira et al. (2015) developed a scale for measuring presenteeism climate, which included three dimensions: (1) extra-time valuation; (2) supervision distrust; and (3) co-workers’ competitiveness. Companies have been increasingly creating climates of presenteeism by stimulating competition from within and by obsessing over productivity increases and organizational development. Recent studies (Mach et al., 2018) indicate that presenteeism climate is related to both the job resources (e.g., supervisor support, job autonomy) and the occurrence of presenteeism behaviors. Another large study on health sector employees in six different countries – Brazil, Ecuador, Lebanon, Portugal, Russia, and Spain – found that presenteeism climate increased WFC and higher levels of WFC were found in non-Latin countries (Ferreira et al., 2019). Despite the absence of recent studies evaluating the role of presenteeism climates during the COVID-19 pandemic, we are convinced that companies that in the past promoted sickness presence at any cost, continue to encourage their sick employees to work remotely when they cannot be present in the organization’s premises (due to confinements). Hence, given the importance of presenteeism climate, we recommend that this construct should be effectively assessed in pandemic contexts and in additional countries (for example, a large-scale study comparing how presenteeism climate is related to the severity of COVID-19 in specific territories).

### Occupational Sectors

The pandemic has affected employees and companies differently depending on the type of sector (Bapuji et al., 2020). Employees from the services sector rely on knowledge work and were easily able to work from home without any (or only marginally reduced) impact on their salary and career. Also, some employees belonging to the gig economy remained unaffected. Inclusively some of them saw an increase in terms of incomes because restaurants and stores were requesting their services to move to the digital and to help them developing new ways of approaching their customers and survive during the confinements.

Conversely, employees in sectors considered as essential, such as frontline jobs, agriculture, construction, food retail, logistics and distribution, public transport, healthcare, and the pharmaceuticals industry suffered from the high risk of exposure to the virus (Bapuji et al., 2020). Most of them worked in precarious conditions with little or no protection and therefore had greater chances of contracting the virus. In such sectors presenteeism is an important behavior to discourage in order to contain COVID-19 outbreaks and protect the health of employees and the entire community. An example of this was the COVID-19 outbreak in an Amazon warehouse (Thomson and Day, 2020). Several employees were infected, and workers protested the alleged hidden cases of sick employees and the silence of middle managers.

## The Societal Context

### Economic Labor Market and Work Regulations

The context of presenteeism is influenced by factors at the societal level (Johns, 2006). Economic factors such as lack of alternative employment options, job insecurity, and limited right to sick pay encourage people to work while sick (Johns, 2010; Lu et al., 2013a; Miraglia and Johns, 2016; Kim et al., 2020). Therefore, to fully understand the COVID-19 lockdown effect on employees’ attendance patterns, researchers should consider the economic, cultural, moral, and social reasons that push employees (such essential workers during the COVID-19 pandemic crises) to attend work despite being exposed to or diagnosed with COVID-19 (Probst et al., 2021).

Both the legislative context (work regulations, social security, and sick-leave coverage) and the current economic labor market conditions (Lohaus and Habermann, 2019) play an important role in explaining why people are turning up for work even though they may be feeling unwell. Thus, the level of development of a country’s welfare system has an important interactive effect with labor market conditions and exerts guidance on which health behaviors are considered acceptable in that specific country (Gustafsson Sendén et al., 2013; Cooper and Lu, 2016). Social health protection including the role, patterns, and costs of paid sick-leave have diverse approaches in different world regions and in different countries (e.g., the paid sick-leave days in Sweden are 9% and in United Kingdom only 3% of the annual working days) (Spasova et al., 2016).

Paid sick-leave performs a crucial role, especially in times of economic crises when many workers fear dismissal and judgment if reporting sick-absence, such as the situation triggered by the pandemic. Low compensation and qualifying days might prevent employees from taking or reporting sick-leave. Therefore, countries with no or limited benefits for paid sick-leave show the lowest number of days lost due to sickness. This includes countries such as the United States, which lacks any national program for paid sick-leave, or the United Kingdom, where no income-related replacement exists (Scheil-Adlung and Sandner, 2010). Such regulations might impact workers’ decisions to continue working while sick.

Other examples of different regulations affecting sickness absence can be found among European countries. The European working conditions surveys (Eurofound, 2012, 2017) report a broad range of indicators that illustrate the differing labor conditions among European countries, which influence and are influenced by the health and safety regulations, and affect people’s wellbeing, productivity, and the occurrence of presenteeism.

Although some labor statistics provide evidence of the different patterns across countries and regions all over the world (e.g., Eurofound, 2012, 2017, 2021; International Labour Organization [ILO], 2012, Working Conditions Laws Database), there are still very few empirical cross-national studies that include the societal and cross-cultural context in their research models.

### The Cross-Cultural Context

Cross-cultural differences and national values play a crucial role in the occurrence of presenteeism (Cooper and Lu, 2016). Among cross-cultural dimensions, the value given to the job well done (e.g., Protestant work ethic), or the shared value of hard work, long hours (Lu et al., 2020), and endurance (Confucian culture), or the perceived legitimacy of absenteeism across cultures (Addae et al., 2013), among others, may play a determinant role in explaining the decision of working during illness. Cross-cultural issues are therefore considered in our health equation.

Country characteristics and culture play a pivotal role in how people react to health conditions and consequently to presenteeism (e.g., Maaravi et al., 2021). Traditionally, studies on presenteeism have focused on two regions – North America and Scandinavia – each entailing a somewhat different research paradigm (Böckerman and Laukkanen, 2010; Johns, 2010). The dominant approach used in the first region is on the productivity losses at work due to presenteeism, whereas in the second region is more often modeled as a lack of job security and risk for future health. The coexistence of these two distinct – and sometimes conflicting – perspectives on presenteeism is essential for a better understanding of its complexities. Johns (2010) sought to connect the two perspectives and bodies of literature into a single, unified theory and also equate presenteeism to absenteeism.

### Poverty and Precarious Work During the Pandemic

The COVID-19 outbreak has highlighted poverty, precarious work, and job inequalities, as it has different effects on individuals and organizations, depending on the type of jobs, social status, or even the level of poverty in the country. In fact, the pandemic made it even more difficult to reach important Sustainable Development Goals (SDGs), such as reducing poverty (SDG1) and achieving decent work (SDG8). Allan et al. (2021) identify three psychological states of work precarity that increased due to the pandemic phenomenon. The first is *precarity of work* and refers to the insecurity about the employee’s continuity of work, which is associated with job, employment, and workplace uncertainty. The second state refers to *precarity at work*, which is associated with the uncertainty in work due to discrimination, harassment, and unsafe working conditions. Workers perceive lack of psychological safety, social rejection, discrimination, and alienation. Finally, *precarity from work* is associated with low salaries, poverty-level wages, perceived income inadequacy, and lack of need satisfaction due to the uncertainty derived from having a job that does not meet the individual’s or family’s basic needs.

Matilla-Santander et al. (2021) identified five important consequences of the COVID-19 crisis among workers in precarious employment: (i) an increased number of precarious jobs; (ii) workers in precarious employment became more precarious; (iii) workers in precarious employment faced more unemployment without being officially laid off; (iv) workers in precarious employment were more exposed to serious stressors and dramatic life changes that may lead to a rise of more infections and diseases; and (v) precarious employment was associated with more uncontrolled contagion and may disrupt or even prevent the control of new COVID-19 outbreaks.

A recent study conducted in Bolivia showed that in the poorest regions the number of deaths in July 2020 were seven times higher than in July 2019. In the richest regions the number of deaths in July 2020 were only two times higher than in July 2019. The economic reality of Bolivia shows that 70% of the Bolivian workforce do not have an employment contract. This evidence justifies that most of the COVID-19 cases (between 40 and 50%) were concentrated in the non-formal economy and specifically with market and transportation workers (Hummel et al., 2021).

Also, in South Africa there was evidence that the poorer employees suffered more because of COVID-19 (and the lockdown). The probability of low-wage earners to lose their jobs during the pandemic outbreak was about eight times higher than high-earner employees. These inequalities increased six times more during COVID-19 over what existed before the pandemic (Nwosu and Oyenubi, 2021). Inequities were even more pronounced among women and regarding race. The COVID-19 pandemic appeared as a catalyst of socioeconomic inequalities in health, including migrant workers, and pejorative actions emerged associated with the phenomena of racism, ethnic minority status, and sexism (Côté et al., 2021).

Working under conditions of economic and legal precariousness (e.g., temporary and unpaid work) in contexts where some companies and sectors (e.g., agriculture, transportation) were facing staffing shortages and service disruptions, led owners to hire precarious workers, which in turn increased the risk of virus transmission among employees, other service users/stakeholders, and their communities (Olding et al., 2021). Similar experiences were documented in the United Kingdom among Roma migrants working in the agriculture sector, where a combination of financial hardship, poverty from work, no access to sick leave, high job insecurity, and discrimination led to high levels of presenteeism during COVID-19 with evident negative implications for individual health and wellbeing (Collins et al., 2021).

During the initial phases of the COVID-19 pandemic workers in general were encouraged to take time off when sick, which was contrary to previous experience in which workers and managers were encouraged to work while ill. However, some precarious workers reported that they did not qualify for sick pay and could not afford to take time off while being ill (The New York Times, 2021). Due to precarious stability and absence of legal protection many employees were also afraid to mention that they were sick and therefore went to work while ill. They were aware that they could lose their jobs and or would not be paid if they had to quarantine. Employees in several contexts hid their symptoms, fearing to be tested and to thereby miss some of their income, or lose it altogether (Loustaunau et al., 2021).

### Models of Presenteeism in the Pandemic Context

The literature shows the negative and positive consequences of people’s decisions to be at work while ill. However, during the COVID-19 pandemic presenteeism was indeed regarded by some managers and employees as something adaptative but also therapeutic and functional (Karanika-Murray and Biron, 2020). Inclusively, managers and co-workers fostered presenteeism cultures (Simpson, 1998; Johns, 2010) and climates (Ferreira et al., 2019) in which being present at work while ill was strongly encouraged. The situation changed with this new pandemic, as the practices implemented in companies all over the world to control and reduce the spread of the virus changed the rules of the game. Specifically, contagious disease appearing as a health condition that (although mentioned) was not properly accounted for in previous presenteeism models, had now been given proper attention. For example, Johns (2010) mentions in his model that normal levels of productivity at work could be affected by acute (e.g., the flu), episodic (e.g., headache), or chronic conditions (e.g., asthma).

**FIGURE 1:**
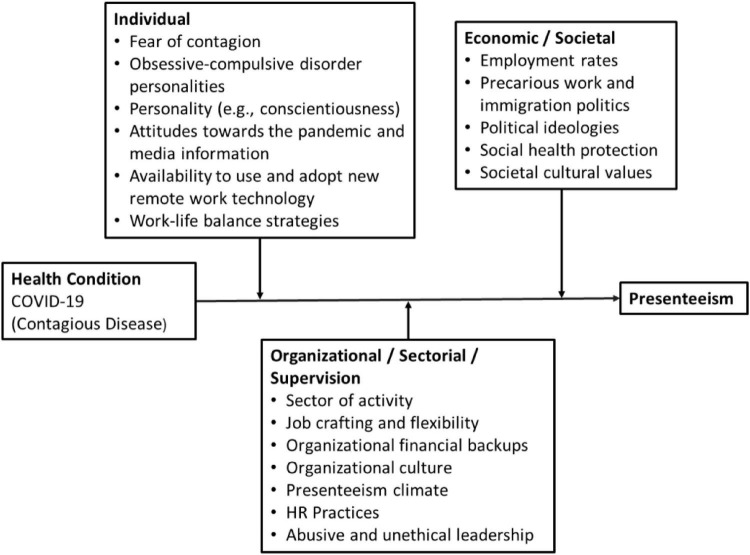
A conceptual model of presenteeism in pandemic context.

Therefore, in the current study we propose a model that seeks to explain how this pandemic crisis placed acute health conditions in the spotlight of managerial practices. Our model accounts for three types of factors: (1) individual (e.g., fear of contagion, personality traits, attitudes toward the media information, availability to use remote technology, and work-life balance); (2) economic/societal (e.g., employment rates, precarious work and immigration politics, political ideologies, health care protection, societal cultural values); and (3) occupational/sectorial/supervision-related factors (e.g., sector of activity, job crafting and flexibility, organizational financial status, organizational culture, organizational climate, HR practices, abusive and unethical leadership). These factors further influence the relationship between an acute and contagious health event such as COVID-19 and presenteeism (see Figure 1).

Regarding individual variables, the literature shows that when employees have greater fear of contagion and perceive health unsafety at work they tend to avoid working while ill (Luksyte et al., 2015). Accordingly, we theorize that employees may have a greater tendency to pressure their supervisors to stay at home and thus to reduce presenteeism in pandemic outbreaks. Moreover, in line with previous studies (see Johns, 2010; Leal and Ferreira, 2021), we propose that personality traits (i.e., obsessive-compulsive disorder personalities, conscientiousness) play an important role in moderating the relationship between acute health conditions in pandemic outbreaks and presenteeism. Specifically, obsessive-compulsive disorder personalities tend to exacerbate the fear of contagion and favor absence behaviors during pandemic contexts (Jalal et al., 2020).

As mentioned by Johns (2010), under normal circumstances (i.e., in the absence of a pandemic), conscientious people might be inclined to attend work while ill. However, we posit that in a pandemic context employees with a clear tendency to be responsible and organized are more inclined to adhere to the institutionalized norms and rules imposed by the national health services and, therefore, stay at home while ill.

Also, even for those employees who do not possess the required skills (nor the equipment) to work remotely (Harford, 2020), the possibility to benefit from the advantages of remote work and family work balance allowed a very substantial reduction in attendance while sick (Darouei and Pluut, 2021; Kim et al., 2021). Therefore, we included in our model the availability to use and adopt new remote work technology and work-life balance strategies as important moderators in the relationship between the health condition and presenteeism behavior.

Along with these individual moderators, economic or societal variables may also play a pivotal role in moderating the relationship between employees’ health conditions in a pandemic context and presenteeism. As discussed above, employees facing job insecurity or working in countries with a lack of alternative employment options (and perhaps coping with fewer rights to receiving a salary while ill) are more prone to presenteeism frequency (Johns, 2010; Lu et al., 2013b; Miraglia and Johns, 2016; Kim et al., 2020).

Also, the economic labor market conditions (i.e., rate of employment) and work regulations, including social security for those who need sick-leave financial support (Lohaus and Habermann, 2019), motivates employees in precarious work conditions to go to work while sick (Hummel et al., 2021). Hence, countries with political ideologies that support the absence of a national program for paid sick-leave (i.e., low social health protection) or impose tight restrictions in terms of immigration policies might influence employees’ decisions to remain working while ill (Scheil-Adlung and Sandner, 2010). Moreover, countries that value job well-done or long hours endurance (Addae et al., 2013) typically promote the occurrence of presenteeism, as discussed above in relation to national cultural values with specific regard to masculine and individualistic values, as well as collectivistic or individualistic cultures.

Our model also considers that the relationship between acute health conditions related to pandemic outbreaks and presenteeism was affected by occupational/sectorial and supervision-related factors. A study in presenteeism among self-employed and organizationally employed in Northwestern Europe (Nordenmark et al., 2019), found that the self-employed reported a significantly higher level of presenteeism than employees working for large companies. This difference is to a high degree explained by the variables measuring time demands, which indicate that the self-employed have a higher risk of reporting presenteeism, as they experience greater time demands. Other research confirms that self-employed individuals, especially self-employed women, report higher levels of time restraints compared to the organizationally employed (Hagqvist et al., 2015). Moreover, in sectors considered essential (e.g., public transport, health care services, pharmaceuticals sector, agriculture, food retail), employees were also more exposed to the virus (Bapuji et al., 2020). Additionally, the lack of flexibility and job crafting in some occupational settings prevented employees from adjusting their workplace to a safer remote-work setting, and thus this situation caused the employees to be more prone to work while ill (Lopes and Ferreira, 2020). The above-mentioned research shows that, even in countries with well-developed social welfare systems, differences in presenteeism across sectors were found within the same country.

Moreover, there is evidence that some companies and supervisors promote presenteeism climates (e.g., Hummel et al., 2021), which tends to pressure employees to work even during the peak of the COVID-19 pandemic and thus to increase the frequency of presenteeism. The HR digital phenomenon was in a certain way accelerated with the COVID-19 pandemic. However, some employees feel pressured by time tracking systems and control mechanisms (Leonardi, 2021) employed by abusive and unethical supervisors (George et al., 2020). This new phenomenon associated to abusive HR practices and unethical behaviors accelerated processes leading to reduced wellbeing (Holman et al., 2002), increased burnout (Cristea and Leonardi, 2019), and WFC (Wang et al., 2021), which can have severe repercussions on attendance behavior and presenteeism.

### The New (Ab)normal Context – Implications for Home and Work Life

The responses of leaders to the volatile, uncertain, complex, and ambiguous (VUCA) conditions imposed by the COVID-19 pandemic outbreak required a collective effort to rethink the meaning of work and implications of leaders’ decisions at the individual, departmental, and organizational levels (Antonacopoulou and Georgiadou, 2021). However, most companies were not ready to take the best advantages of remote work (for all parties involved: employees, departments, and the companies themselves). This new (ab)normal context created by the pandemic introduced new routines and habits and, as a result, new challenges to human resource managers – essentially, the need to introduce more support mechanisms for employees’ wellbeing.

Drawing upon the event system theory (Morgeson et al., 2015), which introduces a conceptual framework in which events appear as a discontinuous and discrete happening that diverges from the stable routines of employees and managers, we are convinced that the COVID-19 outbreak amounts to a profound event affecting the way people live and work. COVID-19 can be considered an enormous social experiment to study how radical and swift the call for work redesign has become, introducing notions such as agility, resilience, and renewal into the mainstream focus not only of business and organizational practices, but of social structures around which “normal” routines were configured (Mukhtar, 2020).

Employees faced profound shifts in their personal lives, interfering with the balance between work and family and the boundaries between the personal and work spheres of life. These changes had momentous social, economic, environmental, and political impacts. Accordingly, it is important to actively study and seek to understand the lessons that the collective responses to the COVID-19 crisis have demanded from managers in general (Antonacopoulou and Georgiadou, 2021) and human resource managers in particular.

The literature has shown *pros* and *cons* regarding the use of remote or distance work with the adoption of new technologies. For example, previous research reports that working from home was less correlated with family work conflict and social isolation during the pandemic outbreak. Moreover, the productivity from those who worked from home was positively related to self-leadership and autonomy (Galanti et al., 2021). Another study revealed that effective supervision (i.e., increased efforts through communication and stronger ties with employees) explained the positive link between remote work activities and organizational performance (Kim et al., 2021). There is also evidence that when employees worked remotely, work was less likely to interfere with the family domain. Employees perceived less WFC and exhaustion levels, thus revealing higher levels of engagement the following morning (Darouei and Pluut, 2021).

In general, there is evidence of emotion trajectories that include the rise and fall of joy toward working from home, and that these ups and downs were influenced by different environment events (Min et al., 2021). The study conducted by Min et al. (2021) showed that stay-at-home government directives affected employees’ transition emotions and their recovery effects. As predicted by Drucker (2012), more than ever supervisors needed to recognize the changes in their subordinates’ lives and adapt their leadership skills to facilitate these emotional and behavioral transitions to a new, unpredicted, and unexpected work model.

Despite the positive benefits (among which we can mention reduced carbon footprints), working from home also brought negative impacts to employees. Personal and professional identities needed to be reconstructed as the boundaries between family and work started to blur. The literature has identified important antecedent risk factors of cardiovascular diseases due to remote work, such as more physical inactivity, social isolation, and loneliness (Sachdeva et al., 2021).

Other studies have reinforced how these detrimental changes could have an impact on non-workday sedentary behavior, poorer sleep quality, an increase in negative mood disorder, reduced perceptions of quality of life, and a considerable decrease in work-related health (Barone Gibbs et al., 2021). Another recent study conducted with higher education scholars revealed that the levels of stress were higher among those who worked remotely several times per week than those working remotely once per month (Heiden et al., 2021). In fact, other empirical studies have suggested that when people combine both daily job demands and daily home demands during remote work, they may experience increased emotional exhaustion (Abdel Hadi et al., 2021).

According to the Conservation of Resources theory (Hobfoll, 1989), employees who perceive a good supply of resources will identify better strategies to cope with the adversities of working from home and have less stress. A study developed by Merino et al. (2021) revealed that the level of stress is dependent upon variables of employment situation, work satisfaction, and the time employees devote to work, as well as the amount of space available in the home and interference from children or other persons there.

Framed on the work-family spillover theory (Staines, 1980), we argue that positive or negative experiences developed in remote work activities can transfer the same positive or negative valences to the home environment. The solution seems to be related with flexibility, as workplace flextime use can decrease employees’ cognitive failures at work and home, because employees are able to increase their levels of perceived control regarding home and work duties more consistently (Hsu et al., 2021).

In general, managers should endeavor to reduce the detrimental relationship between job and home demands with emotional exhaustion. Findings from previous studies suggest that a supportive organizational culture (e.g., open communication, empowerment, teamwork, and participation) can generate positive spillover effects on employees (Sok et al., 2014). It is vital to diagnose each employee’s needs regarding remote work considering different aspects. For example, employees living alone may have very different virtual working demands when compared to employees living with children or others. The supervisor profile must be redefined to face the challenges in motivating employees in distant or virtual working contexts. Moreover, HR professionals must adjust their training proposals, performance appraisals systems, incentives, and occupational health support (Kniffin et al., 2021).

### The New (Ab)normal – Remote-Work Presenteeism Behavior

As mentioned above, more than ever companies are adopting remote work, essentially those in which their employees assessed their remote work practices as positive (Espitia et al., 2021). Remote working can be used to the advantage of the employer. For example, companies such as Twitter see remote work as a possibility to reduce costs (Kresge, 2021). Moreover, we assume that countries, regions, and companies with masculine and individual cultures (Martinez et al., 2018) may have a greater tendency to promote remote work when their employees are sick. Also, companies that value long hours at work and stimulate highly competitive environments (e.g., Simpson, 1998) would be more prone to adopt remote work as a new way of presenteeism. Accordingly, with the advance and use of new remote work technologies during the pandemic, being sick will no longer be a sufficient “excuse” not to complete tasks according to some companies and supervisors. In certain cases, employees would no longer have the opportunity to recover at home from acute, episodic, or even chronic health conditions – they would now be compelled to complete their tasks (while at home). Therefore, herein we conceptualize the appearance of a new construct named remote-work presenteeism behavior, in which employees are invited to stay at home and work remotely while being ill. This conceptualization draws on previous studies that emphasize the possibility of presenteeism in domestic work activities, affecting both male and female partners’ organizational productivity while ill (Leal and Ferreira, 2021).

**FIGURE 2:**
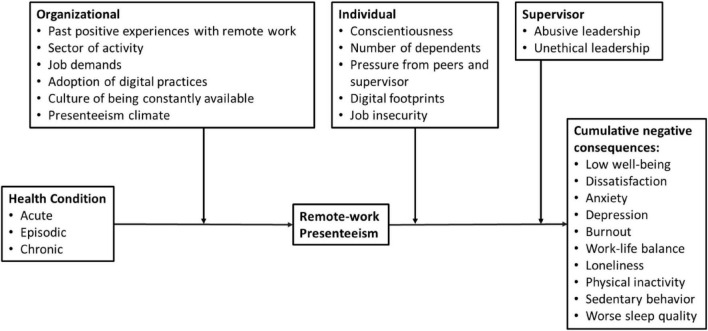
Remote-working presenteeism behavior model.

In Model 2 (see Figure 2), we conceptualize a framework in which individuals with different health conditions (i.e., acute, episodic, or chronic) are “invited” to develop remote work presenteeism. This behavior is influenced by several organizational variables (e.g., past positive experience with remote work, sector of activity, adoption of digital practices, cultures of being permanently available, and presenteeism climate).

Companies with presenteeism climate stimulate competition among employees and develop extra-time valuation, and employees perceive that their supervisors do not trust them when they mention that they are having a health problem (Ferreira et al., 2015). This behavior has serious consequences, and employees tend to be more pressured and tempted to develop presenteeism remote work. Additionally, companies that develop cultures of being permanently available (Simpson, 1998), whereby managers could adopt new digital practices and use time tracking systems, continuous communication channels, and intrusive surveillance devices (George et al., 2020) may also create implications for presenteeism remote work behaviors. These requirements could promote a perception of increased job demands associated with a culture of being always available (Parker et al., 2020), thus generating more pressure by increasing anxiety, depression, and job dissatisfaction (Holman et al., 2002).

During the COVID-19 outbreak, several companies around the world made huge investments in remote work technologies and new occupational norms regulating attendance in specific sectors (e.g., services; gig economy; self-employees). This circumstance, in our opinion, could motivate employees to work at home while ill (Bapuji et al., 2020).

Our model also shows that remote-work presenteeism and cumulative negative consequences appear as a consequence of several supervisor and individual characteristics. Concerning individual variables, Johns (2010) states that employees with conscientiousness personality traits might be inclined to go to work while ill. In fact, conscientious individuals may show perseverance in the face of adversity and strong work ethic values that encourage them to develop presenteeism remote-work behaviors and thus enter a spiral of negative work outcomes associated with productivity losses due to illness. This could be even more reinforced in contexts in which working remotely while ill occurs while surrounded by dependents such as children or elderly parents. Moreover, when employees recognize that the company has their daily activities under scrutiny, they may perceive high job insecurity and perceive low well-being, dissatisfaction, anxiety, and burnout (Cristea and Leonardi, 2019).

While working remotely with a health problem, the absence of social support (from peers and supervisor) and high job demands imposed by abusive and unethical leadership (both at the supervisory level) may lead to physical inactivity, social isolation, low work-life balance, procrastination, and loneliness (Sachdeva et al., 2021; Wang et al., 2021). We therefore conceptualize a model in which remote-work presenteeism may lead to cumulative negative consequences (e.g., low well-being, anxiety, depression, burnout, loneliness, sedentary behavior, and poorer sleep quality), and that these consequences may be reinforced when associated with individual, supervisory, and organizational variables mentioned above and depicted in Figure 2.

## Conclusion

With the emergence of COVID-19, pandemic researchers developed theoretical conceptual frameworks to understand how to cope with the undesirable consequences and promote wellbeing (e.g., Ramkissoon, 2020). This paper highlights the emerging trends in the field of presenteeism following the increased use of digitalization and the fast shift to remote work that the COVID-19 pandemic has accelerated everywhere. We reviewed organizational determinants (i.e., changes in the workplace, presenteeism climate), occupational sector differences in presenteeism, and the societal context (i.e., legislative, employment, economic conditions, and cultural values). This allows us to broaden the scope and consider not only the individual determinants of the act of presenteeism behavior (Johns, 2010), but also the social determinants of attendance at work during the COVID-19 pandemic times. In our opinion, these findings constitutes and advancement in the event system theory (Morgeson et al., 2015) as it explains how organizations and individuals (motivated by the COVID-19 pandemic) changed their concept of work while ill. We integrated all these factors in a multi-level model that looks at the relationships between an acute health condition (i.e., COVID-19 contagious disease) and the choice between absenteeism or presenteeism, by accounting for individual, organizational, managerial, economic, and societal factors, to shed light on the behavior of presenteeism at work. Furthermore, due to the remote-working presenteeism behavior developed during the pandemic lockdown, whereby employees remain at home but feel pressure to continue working virtually while being sick (Sachdeva et al., 2021; Wang et al., 2021), we have proposed a second multi-level model to capture this new normality, and the potential cumulative negative consequences that remote-work presenteeism could have for individuals and organizations. Based on our findings, we are strongly convinced that governments, policymakers, managers, and healthcare professionals should introduce regulations and interventions for employees to deliver better equipped people to cope in the post-pandemic world (Ramkissoon, 2021). We invite scholars and practitioners to push forward these contributions to the presenteeism field by considering the different angles and the different levels of analyses of the phenomenon, as well as longitudinal research designs, cross country, and between sector comparisons – with the goal of better capturing the new patterns of attendance at work with the exponential implementation of digitalization and remote working practices. Lastly, we encourage researchers to test empirically the proposed presenteeism models to understand how this new remote-work presenteeism behavior and this apparently “new (ab)normality” may bring negative consequences for individuals and organizations.

## Data Availability Statement

The original contributions presented in the study are included in the article, further inquiries can be directed to the corresponding author.

## Author Contributions

All authors listed have made a substantial, direct, and intellectual contribution to the work, and approved it for publication.

## Conflict of Interest

The authors declare that the research was conducted in the absence of any commercial or financial relationships that could be construed as a potential conflict of interest.

## Publisher’s Note

All claims expressed in this article are solely those of the authors and do not necessarily represent those of their affiliated organizations, or those of the publisher, the editors and the reviewers. Any product that may be evaluated in this article, or claim that may be made by its manufacturer, is not guaranteed or endorsed by the publisher.
